# Dynamic control of *ERG9* expression for improved amorpha-4,11-diene production in *Saccharomyces cerevisiae*

**DOI:** 10.1186/s12934-015-0220-x

**Published:** 2015-03-18

**Authors:** Jifeng Yuan, Chi-Bun Ching

**Affiliations:** Department of Chemical and Biomolecular Engineering, National University of Singapore, 4 Engineering Drive 4, Singapore, 117585 Singapore; Synthetic Biology Research Consortium, National University of Singapore, 28 Medical Drive, Singapore, 117456 Singapore; Singapore Institute of Technology, 10 Dover Drive, Singapore, 138683 Singapore

**Keywords:** Mevalonate pathway, Dynamic control, *ERG9*, Ergosterol-responsive promoter, Isoprenoids, *Saccharomyces cerevisiae*

## Abstract

**Background:**

To achieve high-level production of non-native isoprenoid products, it requires the metabolic flux to be diverted from the production of sterols to the heterologous metabolic reactions. However, there are limited tools for restricting metabolic flux towards ergosterol synthesis. In the present study, we explored dynamic control of *ERG9* expression using different ergosterol-responsive promoters to improve the production of non-native isoprenoids.

**Results:**

Several ergosterol-responsive promoters were identified using quantitative real-time PCR (qRT-PCR) analysis in an engineered strain with relatively high mevalonate pathway activity. We found mRNA levels for *ERG11*, *ERG2* and *ERG3* expression were significantly lower in the engineered strain over the reference strain BY4742, indicating these genes are transcriptionally down-regulated when ergosterol is in excess. Further replacement of the native *ERG9* promoter with these ergosterol-responsive promoters revealed that all engineered strains improved amorpha-4,11-diene by 2 ~ 5-fold over the reference strain with *ERG9* under its native promoter. The best engineered strain with *ERG9* under the control of P_*ERG1*_ produced amorpha-4,11-diene to a titer around 350 mg/L after 96 h cultivation in shake-flasks.

**Conclusions:**

We envision dynamic control at the branching step using feedback regulation at transcriptional level could serve as a generalized approach for redirecting the metabolic flux towards product-of-interest.

## Background

Microbial production of natural products in genetically tractable microbes has gained tremendous interest in the recent years. In order to produce these molecules at industrial levels, pathway genes involved in the synthesis of these molecules must be expressed at appropriately balanced levels, to avoid the accumulation of toxic intermediates or bottlenecks that result in growth inhibition or suboptimal yields [1-3]. Moreover, it often requires the metabolic flux towards side pathways to be minimized or completely eliminated [4-8]. For example, high-level production of non-native isoprenoid products requires the metabolic flux to be diverted from the production of ergosterol to the heterologous metabolic reactions. Down-regulation of *ERG9* gene which encodes squalene synthase (the first committed step after farnesyl diphosphate in ergosterol biosynthesis), using the methionine-repressible *MET3* promoter or copper-repressible *CTR3* promoter, increased amorpha-4,11-diene production an additional 2-fold [9,10]. Other approaches such as harnessing weak promoter for controlling *ERG9* expression and utilizing *HXT1* promoter to couple *ERG9* expression with glucose concentration also showed promising results [4,11].

**Figure 1:**
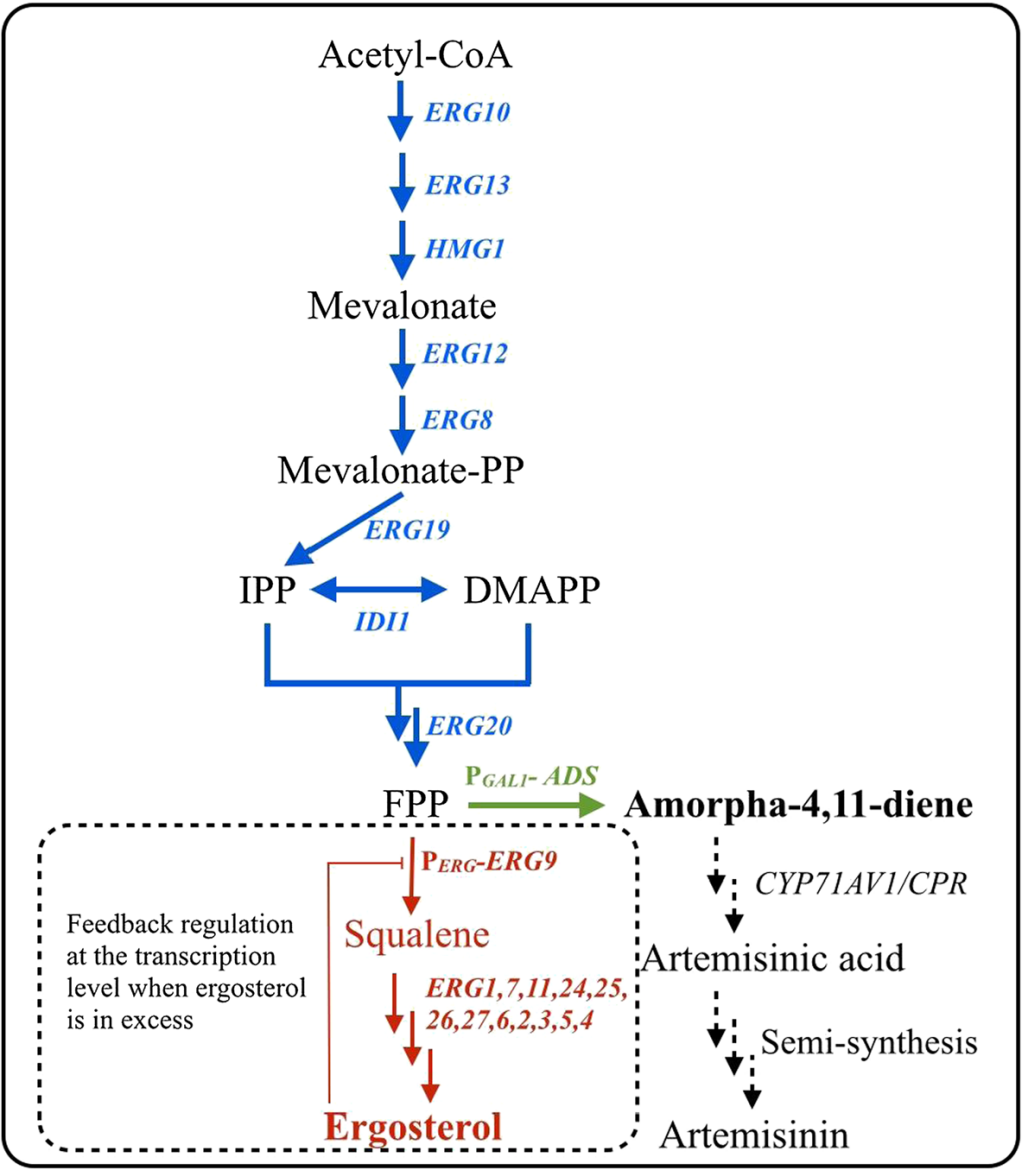
**Schematic diagram of ergosterol-responsive promoters for controlling the metabolic flux towards erogosterol biosynthesis pathway.** Genes from the mevalonate pathway in *S. cerevisiae* are shown in blue; heterologous expression of *ADS* gene is shown in green; and genes from ergosterol biosynthesis pathway are shown in red. The *ERG9* expression is put under the control of ergosterol-responsive promoter (P_*ERG*_) to achieve dynamic control of squalene synthase availability in response to intracellular ergosterol levels. The pathway intermediates IPP, DMAPP and FPP are defined as isopentenyl pyrophosphate, dimethylallyl pyrophosphate and farnesyl diphosphate, respectively.

As ergosterol fulfills several essential functions and each requires optimal sterol concentrations, synthesis of sterols in yeast is tightly regulated. In budding yeast, it requires thirteen-enzymatic steps to synthesize ergosterol from farnesyl diphosphate – a precursor from the mevalonate pathway (Figure 1). Previously, *ERG9* expression in yeast was reported to be positively and negatively regulated by diverse factors such as the heme activator protein transcription factor HAP1/2/3/4 and the phospholipid transcription factor complex INO2/4 [12,13]. Sterol biosynthetic mutations at *ERG3*, *ERG7* and *ERG24* also increased *ERG9* expression level. However, naturally occurring cognate regulator for *ERG9* expression will rarely suffice to regulate an engineered pathway with higher metabolic flux. This leads us to search for other ergosterol-responsive promoters from ergosterol biosynthesis pathway (Table 1) for a better and tighter control of *ERG9* expression, with further improved production of non-native isoprenoids. Previous investigations revealed that genes involved in ergosterol biosynthesis pathway such as *ERG1, ERG11, ERG2* and *ERG3* were transcriptionally up-regulated when yeast cells are treated with inhibitors to restrict metabolic flux towards ergosterol biosynthesis [14-16]. Therefore, these ergosterol-responsive promoters can be interesting candidates for the dynamic regulation of *ERG9* expression in budding yeast.

**Table 1:** **List of genes involved in the mevalonate and ergosterol biosynthesis pathways**

**Gene**	**Discription**
The mevalonate biosynthesis pathway	
*ERG10*	Acetyl-CoA C-acetyltransferase (EC:2.3.1.9)
*ERG13*	Hydroxymethylglutaryl-CoA synthase (EC:2.3.3.10)
*HMG1/2*	Hydroxymethylglutaryl-CoA reductase (EC:1.1.1.34)
*ERG12*	Mevalonate kinase (EC:2.7.1.36)
*ERG8*	Phosphomevalonate kinase (EC:2.7.4.2)
*ERG19*	Diphosphomevalonate decarboxylase (EC:4.1.1.33)
*IDI1*	Isopentenyl-diphosphate delta-isomerase (EC:5.3.3.2)
*ERG20*	Bifunctional (2E,6E)-farnesyl diphosphate synthase/dimethylallyltranstransferase (EC:2.5.1.10)
The ergosterol biosynthesis pathway	
*ERG9*	Squalene synthase (EC:2.5.1.21)
*ERG1*	Squalene monooxygenase (EC:1.14.13.132)
*ERG7*	Lanosterol synthase (EC:5.4.99.7)
*ERG11*	Lanosterol demethylase (EC:1.14.13.70)
*ERG24*	C-14 sterol reductase (EC:1.3.1.70)
*ERG25*	Methylsterol monooxygenase (EC:1.14.13.72)
*ERG26*	Sterol-4-alpha-carboxylate 3-dehydrogenase, decarboxylating (EC:1.1.1.170)
*ERG27*	3-keto-steroid reductase (EC:1.1.1.270)
*ERG6*	Sterol 24-C-methyltransferase (EC:2.1.1.41)
*ERG2*	C-8 sterol isomerase (EC:5.3.3.5)
*ERG3*	C-5 sterol desaturase (EC:1.3.3.-)
*ERG5*	C-22 sterol desaturase (EC:1.14.-.-)
*ERG4*	C24(28) sterol reductase (EC:1.3.1.71)

Here, we investigated mRNA levels of *ERG1*, *ERG11*, *ERG2* and *ERG3* in an engineered yeast with relatively high mevalonate pathway activity [3]. Among these candidates, we found *ERG11*, *ERG2* and *ERG3* were transcriptionally down-regulated in our engineered strain when compared to the reference strain of BY4742, whereas mRNA level of *ERG1* gene in both strains remained relatively low. When the engineered strains with *ERG9* under the control of different ergosterol-responsive promoters were examined for the production of amorpha-4,11-diene (Figure 1), strains showed 2 ~ 5-fold higher levels of amorpha-4,11-diene than the reference strain. Among them, P_*ERG1*_ showed the best result for improving amorpha-4,11-diene production yielding a final titer around 350 mg/L after 96 h cultivation in 250 mL shake-flasks. We envision dynamic control using side-product regulated systems could serve as an attractive strategy for redirecting metabolic flux towards product-of-interest. The methodology described here would be generalizable for engineering other metabolic pathways.

## Results

### Characterization of ERG1, ERG11, ERG2 and ERG3 expression levels in the engineered strains with high mevalonate pathway activity

As ergosterol fulfills several essential functions that require optimal sterol concentrations, synthesis of sterols in yeast must be tightly regulated. For example, squalene epoxidase (encoded by *ERG1*) is an essential enzyme in the ergosterol-biosynthesis pathway and catalyzes the squalene epoxidation step. Inhibition of ergosterol biosynthesis with the antifungal drug terbinafine at squalene epoxidase step can trigger increased level of *ERG1* expression in a concentration-dependent manner to a maximum of sevenfold [15]. Inhibition of a later step in the ergosterol biosynthetic pathway by ketoconazol, an inhibitor of the lanosterol-14*α*-demethylase (encoded by *ERG11*), also induces the expression of *ERG1*, indicating that *ERG1* expression is positively regulated by diminished intracellular ergosterol levels. Similarly, various other ergosterol biosynthetic pathway genes such as *ERG11* [17,18], *ERG2* [16] and *ERG3* [14] are transcriptionally up-regulated when ergosterol biosynthesis is inhibited using different drugs. Therefore, it is likely that ergosterol-responsive promoters – by definition – will respond to excessive amounts of intracellular ergosterol and trigger the down-regulation of corresponding genes accordingly.

**Figure 2:**
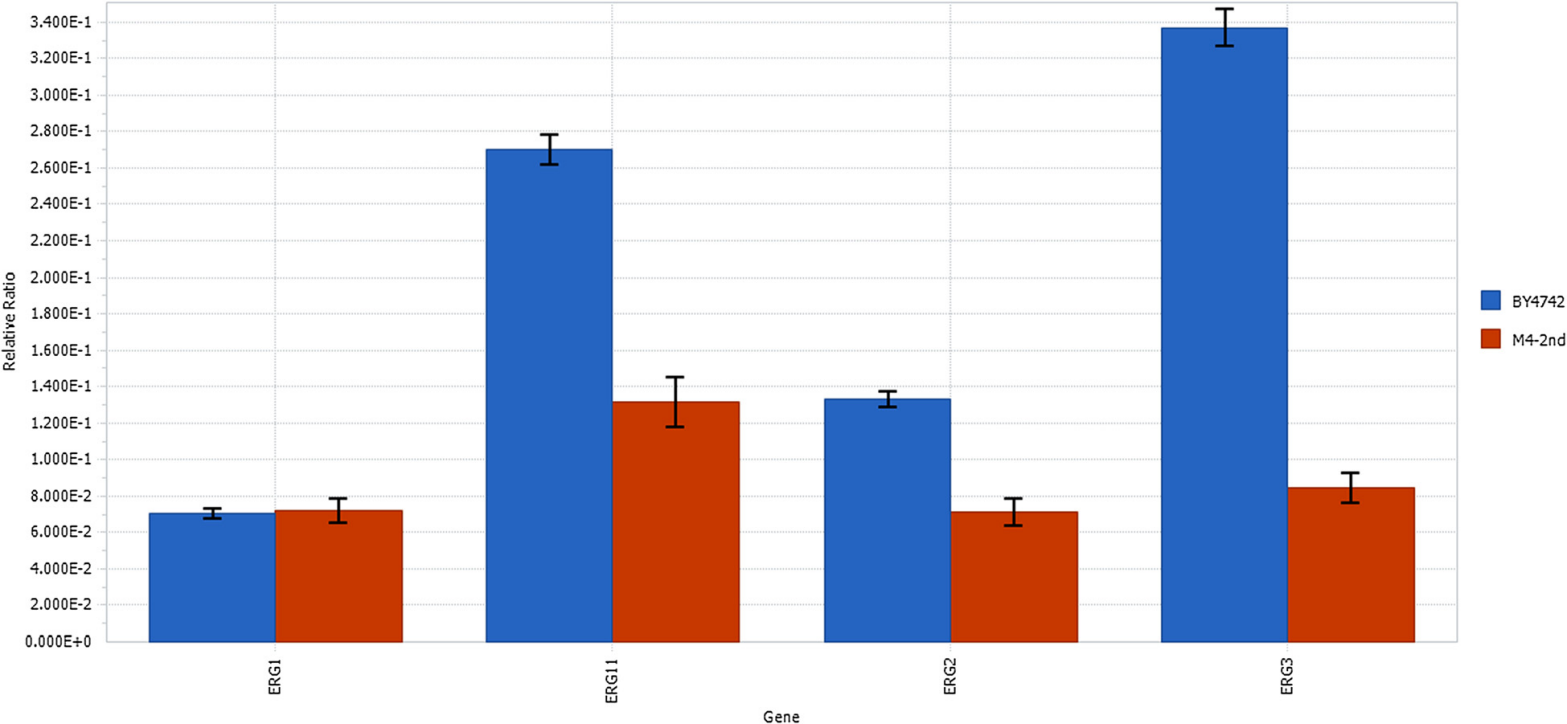
**qRT-PCR analysis of** ***ERG1*** **,** ***ERG11*** **,** ***ERG2*** **and** ***ERG3*** **in an engineered strain with high mevalonate pathway activity over the reference strain BY4742.** Two cultures of each strain, BY4742 (Control) and M4-2nd, were inoculated in SC media at initial OD_600_ of 0.05 and harvested at early-log phase for qRT-PCR analysis. The results are presented as the relative abundances of *ERG1*, *ERG11*, *ERG2* and *ERG3* in each strain with respect to *ACT1*. Values represented the average and standard deviation of three independent experiments.

In the present study, we decided to investigate whether the abovementioned genes, namely, *ERG1*, *ERG11*, *ERG2* and *ERG3*, are transcriptionally downregulated in the engineered strain with relatively high mevalonate pathway activity. Previously, our group has successfully engineered yeast strains with significant improvement of mevalonate pathway activity for high-level production of amorpha-4,11-diene. Notably, we found that strain M4-2nd without *ADS* gene showed extremely slow growth profile as compared to BY4742 when cultivated in galactose medium (data not shown) suggesting that the accumulation of intermediates such as DMAPP, IPP and FPP might be toxic to the yeast cells, as they are in *E. coli* [19]. Therefore, strain M4-2nd expressing *ADS* gene was used for mRNA extraction in the induction medium. When comparing the mRNA abundance of *ERG1*, *ERG11*, *ERG2* and *ERG3* in the engineered strain M4-2nd over the reference strain BY4742, we found *ERG11*, *ERG2* and *ERG3* did show a sharp decrease of mRNA levels in the engineered strain M4-2nd (Figure 2). Intriguingly, there was no obvious change for *ERG1* expression levels between the reference strain and the engineered strain (Figure 2), and the mRNA abundances of *ERG1* in both strains were relatively low, which may also explain the accumulation of large amount of squalene in an engineered yeast with deregulated expression of HMG-CoA reductase, whereas the sterol contents showed only small changes [18].

### Amorpha-4,11-diene production in engineered strains with ERG9 under the control of different ergosterol-responsive promoters

As different transcripts may have varying half-lives, the relative strengths of abovementioned ergosterol-responsive promoters are not reflected by qRT-PCR analysis as shown in Figure 2. The use of reporter genes such as *lacZ* under different ergosterol-responsive promoters may help determine the promoter strengths, but it will be too cumbersome to systematically evaluate the relative promoter strengths during different growth phases. Therefore, upon successful demonstration of transcriptional down-regulation of ergosterol biosynthesis genes in the engineered strain with high mevalonate pathway activity, we sought to directly test our engineered strains with *ERG9* under the control of different ergosterol-responsive promoters for amorpha-4,11-diene production [20].

**Figure 3:**
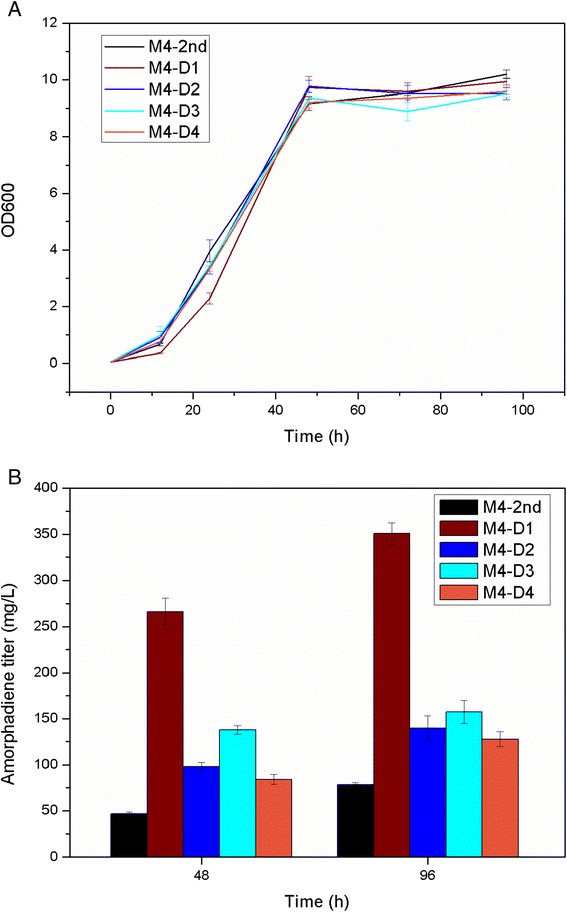
**Growth profile (A) and amorpha-4,11-diene production (B) in engineered strains.** Strains M4-D1 ~ D4 with *ERG9* under the control of different ergosterol-responsive promoters were transformed with plasmid pRS415ADS for producing amorpha-4,11-diene. Here, strain M4-D1 ~ D4 are derived from strain M4-2nd with *ERG9* under the control of P_*ERG1*_, P_*ERG11*_, P_*ERG2*_ and P_*ERG3*_, respectively. Strain M4-2nd with plasmid pRS415ADS was used as control. Strains were cultivated in 250 mL flasks supplemented with 25 mL SC-LEU media. The amorpha-4,11-diene production in engineered strains was measured using GC-FID after 48 h and 96 h of cultivation. Values represented the average and standard deviation of three independent experiments.

**Figure 4:**
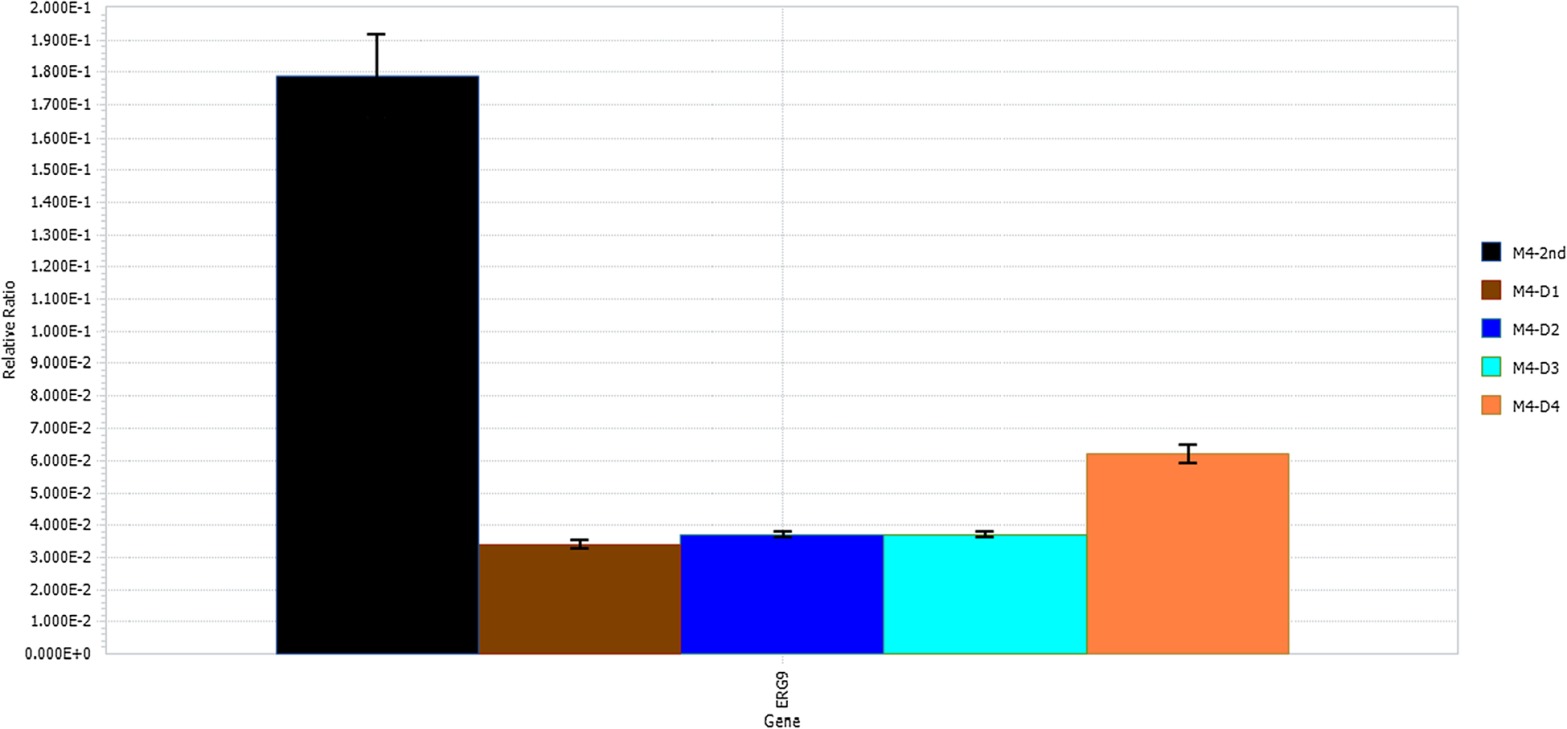
**qRT-PCR analysis of** ***ERG9*** **expression in the engineered strains.** Five cultures of each strain, M4-2nd, M4-D1, M4-D2, M4-D3 and M4-D4 were inoculated in SC media at initial OD_600_ of 0.05 and harvested during early-log phase for qRT-PCR analysis. All strains harboring plasmid pRS415ADS were subjected to qRT-PCR analysis to determine *ERG9* expression levels in engineered strains. The results are presented as the relative abundances of *ERG9* in each strain with respect to that of *ACT1*. Values represented the average and standard deviation of three independent experiments.

In the present study, all engineered strains with *ERG9* under different ergosterol-responsive promoters were transformed with pRS415ADS to evaluate the effect of dynamic control of *ERG9* expression on amorpha-4,11-diene production. The resulting strains were designated as M4-D1, M4-D2, M4-D3, and M4-D4 with the *ERG1*, *ERG2*, *ERG3*, and *ERG11* promoters to drive *ERG9* expression, respectively (Table 2). Interestingly, all engineered strains with *ERG9* under different ergosterol-responsive promoters improved the amorpha-4,11-diene titer substantially, with up to a 5-fold improvement over the reference strain, which contains *ERG9* under its native promoter (Figure 3B). Among these strains, strain M4-D1 showed the best result at 5-fold improvement and produced amorpha-4,11-diene to a final titer around 350 mg/L after 96 h cultivation in shake flasks, suggesting that *ERG9* under the control of P_*ERG1*_ most efficiently restricted metabolic flux towards ergosterol biosynthesis. Moreover, there was an inverse correlation of amorpha-4,11-diene levels to growth rate during the early exponential phase (Figure 3A). As these ergosterol-responsive promoters can respond to the diminished level of ergosterol and compensate with elevated transcription levels [14,15,17], the perturbation of growth rate might be caused by the accumulation of toxic intermediates, as seen in *E. coli* [19]. To further confirm that the improved amorpha-4,11-diene titer in strains M4-D1 ~ D4 was attributed to the regulation of *ERG9* expression, we next sought to systematically compare the mRNA abundance of *ERG9* in all engineered strains. As can be seen from Figure 4, qRT-PCR analysis revealed that mRNA levels of *ERG9* in strains M4-D1 ~ D4 were significantly lower when compared to the reference strain M4-2nd, indicating that the improvement of amorpha-4,11-dien levels was caused by *ERG9* restriction using ergosterol-repsonsive promoters. Collectively, our findings suggested dynamic control of *ERG9* expression using ergosterol-responsive promoters could be an alternative strategy for improving isoprenoid productions. Future work will be focusing on optimizing the dynamic-sensor device and FPP consumption module to allow the consumption of intermediates at the appropriate rates for optimal pathway activities.

**Table 2:** **List of plasmids and strains used in the present study**

**Name**	**Description**	**References**
Plasmid name		
pUC18	Plasmid for cloning in *E. coli*	Invitrogen
pUG72	Plasmid harboring *URA3* selection marker	[30]
pSH68	Plasmid harboring *Cre* gene under the control of P_*GAL1*_	[30]
pRS425ADS	pRS425::P_*GAL1*_-*ADS*-T_*CYC1*_	[9]
pRS415ADS	pSH68 derivative with pRS415::P_*GAL1*_-*ADS*-T_*CYC1*_	This study
pURA3-Blank	pUC18 derivative containing *URA3* selection marker from pUG72	This study
pURA3-ERG1p	pURA3-Blank derivative with insertion of promoter region from *ERG1* gene	This study
pURA3-ERG11p	pURA3-Blank derivative with insertion of promoter region from *ERG11* gene	This study
pURA3-ERG2p	pURA3-Blank derivative with insertion of promoter region from *ERG2* gene	This study
pURA3-ERG3p	pURA3-Blank derivative with insertion of promoter region from *ERG3* gene	This study
Strain name		
M4-2nd	BY4742 derivative with the relatively high mevalonate pathway activity	[3]
M4-D1	M4-2nd with *ERG9* under the control of P_*ERG1*_	This study
M4-D2	M4-2nd with *ERG9* under the control of P_*ERG11*_	This study
M4-D3	M4-2nd with *ERG9* under the control of P_*ERG2*_	This study
M4-D4	M4-2nd with *ERG9* under the control of P_*ERG3*_	This study
M4-2nd-L	Strain M4-2nd harboring plasmid pRS415ADS	This study
M4-D1-L	Strain M4-D1 harboring plasmid pRS415ADS	This study
M4-D2-L	Strain M4-D2 harboring plasmid pRS415ADS	This study
M4-D3-L	Strain M4-D3 harboring plasmid pRS415ADS	This study
M4-D4-L	Strain M4-D4 harboring plasmid pRS415ADS	This study

## Discussion

Dynamic regulation would allow an organism to adapt its metabolic flux to changes within the host or in its environment [21], which would allow the delivery of intermediates at the appropriate levels and rates for optimal pathway activities. In one of the pioneering examples of dynamic regulation system, acetyl phosphate was used as an indirect indicator for excess amount of glucolytic flux to regulate the heterologous lycopene biosynthesis pathway [22]. Recently, Zhang *et al.* demonstrated that dynamic regulation using FadR-based sensing device in *E. coli* could improve the FAEE production by 3-fold to 28% of theoretical maximum and also substantially improve the stability of biodiesel-producing strains [23]. In a more recent effort, Dahl *et al.* exploited the stress-response promoter system to dynamically control the mevalonate biosynthesis pathway in *E. coli* and the resulting strain showed 2-fold improvement of amorpha-4,11-diene production compared to either a constitutive expression system or an IPTG inducible system [24]. Nature has provided abundant ligand-responsive transcription factors, whose DNA-binding activities are regulated by various types of molecules, including nucleic acids, carbohydrates, lipids, amino acids and many secondary metabolites [23]. For example, tyrR transcriptional factor from *E. coli* was reported to play an important role for regulating L-tyrosine biosynthesis pathway [25] and those tyrR-mediated tyrosine-responsive promoters may be used for dynamically controlling the side-pathway expressions and improving the product titer of tyrosine-derived compounds such as alkaloids.

High-level production of product-of-interest in microbial hosts also requires the metabolic flux towards competing pathways to be minimized or completely eliminated if it is not essential for the cells to survive [4-8]. However, many competing pathways inside the cell are producing compounds that are essential to support cell growth. For example, isoprenoid biosynthesis pathway is not only essential for ergosterol production to maintain proper membrane structure, but also for heme A and ubiquinone biosynthesis. Deletion of *ERG9* gene to block metabolic flux towards biosynthesis of these essential components would result in inviable strains. Currently, researchers have explored the methionine-repressible *MET3* promoter or copper-repressible *CTR3* promoter for restricting *ERG9* expression to divert metabolic flux from ergosterol biosynthesis to non-native isoprenoid production, which improved amorpha-4,11-diene production an additional 2-fold [9,10]. Other approaches such as harnessing a weak *CYC1* promoter for controlling *ERG9* expression or using a *HXT1* promoter to couple *ERG9* expression with glucose concentration also showed exciting results [4,11]. Here, we demonstrated that dynamic control of *ERG9* expression using ergosterol feedback regulation mechanism could also substantially improve the amorpha-4,11-diene titer. As sterols fulfill several essential functions inside the cell, insufficient flux towards ergosterol and other sterols caused by restricting *ERG9* expression was reported to cause deleterious effect on cell growth [6]*.* In this case, the amount and the timing of adding repressors such as copper sulfate and methionine to the broth during different growth phases was systematically investigated to avoid growth inhibition and to achieve the optimal product titer. In contrast, our dynamic control device harnesses ergosterol-responsive promoters to adjust *ERG9* expression according to the cell’s need of ergosterols; the sterol level can be always maintained at an appropriate level for optimal cell growth under changing environments (Figure 1). Once the cell senses the excess of ergosterol, *ERG9* expression under the control of these ergosterol-responsive promoters will be tuned down, which will result in redirection of the metabolic flux towards non-native isoprenoid production. Another benefit of ergosterol-regulated system for dynamic control of *ERG9* expression is that it also eliminates the requirement of adding repressors such as copper sulfate or methionine, which will significantly simplify the fermentation process, as well as reduce production costs.

In the future, promoter engineering using error-prone PCR can be further explored to improve the sensitivity and dynamic range of these ergosterol-responsive promoters, and to achieve even tighter control of gene expression. Alternatively, hybrid promoter systems may also be developed by fusion of ergosterol-responsive elements with other well-studied promoters. Furthermore, degradation signal through N-End rule [26,27] may be further used to modulate the enzyme turnover rate for a more robust and accurate dynamic control device.

## Conclusions

In summary, a dynamic control device using side-product regulated system offers an alternative strategy to conventional approaches, such as gene deletion or *CTR3*/*MET3* repressible promoters, for restricting metabolic flux towards side-product biosynthesis. The methodology described here would serve well as a generalized technique for engineering additional metabolic pathways.

## Methods

### Strains, plasmids and reagents

*Escherichia coli* strain DH5α was used for general plasmid constructions and the strain was cultivated at 37°C in Luria-Bertani medium with 100 μg/mL ampicillin. Previously engineered *S. cerevisiae* strain M4-2nd with high mevalonate pathway activity was used as the parental strain for all yeast strain constructions. This strain was cultured in rich YPD medium. Engineered strains with different auxotrophic selection markers were grown in synthetic complete (SC) medium with leucine or uracil dropout where appropriate. For induction of genes under the control of galactose inducible promoters, *S. cerevisiae* strains were grown in 1.8% galactose plus 0.2% glucose. Plasmid pSH68 and pUG72 were obtained from EUROSCRAFF. Plasmid pRS425ADS with the codon optimized amorpha-4,11-diene synthase (ADS) gene from the plant *Artemisia annua* [9] was kindly provided by Prof. Jay Keasling from University of California, Berkeley. Restriction enzymes, Taq polymerase, alkaline phosphatase (CIP) and T4 ligase were purchased from New England Biolabs (Beverly, MA). iProof HF polymerase and iScript^TM^ Reverse Transcription Supermix were obtained from BioRad (Hercules, CA). Gel extraction kit, PCR purification kit, Plasmid purification kit and RNeasy Mini Kit were purchased from QIAGEN (Hilden, Germany). FastStart Essential DNA Green Master Mix was purchased from Roche (Singapore, SG). All of the chemicals used in this study were purchased from Sigma-Aldrich (St. Louis, MO).

### Plasmid construction and yeast transformation

Oligonucleotides used for plasmid construction are listed in Table 3. To create the genome integration cassette, a series of plasmids was constructed as follows. Firstly, *Kluyveromyces lactis URA3* selection marker was amplified from plasmid pUG72 using primer pair F_URA3_*Hin*dIII/R_URA3_BE. The PCR product with size around 1.4 kb was purified, digested with *Hin*dIII/*Eco*RI and inserted into pUC18 cut with the same enzyme pair, to yield pURA3-Blank. Next, the 801 bp promoter region of *ERG1* was amplified from genomic DNA of *S. cerevisiae* BY4742 using primer pair F_ERG1p_*Bam*HI/R_ERG1p_*Eco*RI. The PCR product was cut with *Bam*HI/*Eco*RI, and inserted into pURA3-Blank cut with *Bam*HI/*Eco*RI, to yield pURA3-ERG1p. Similarly, the 1000 bp promoter region of *ERG11*, the 807 bp promoter region of *ERG2*, and the 802 bp promoter region of *ERG3* were PCR amplified from genomic DNA of *S. cerevisiae*, cut with *Bam*HI/*Eco*RI, and inserted into pURA3-Blank cut with *Bam*HI/*Eco*RI, to yield plasmid pURA3-ERG11p, pURA3-ERG2p and pURA3-ERG3p, respectively (Table 2). To this end, these plasmids served as template for the amplification of genome integration cassette using primer pair F_ERG9p_Int/R_ERG9p_Int.

**Table 3:** **Oligonucleotides used for constructing plasmids and qPCR studies**

**Name**	**Discription**
F_URA3_*Hin*dIII	AGAGA*AAGCTT*GCAGCGAGAACACGACCACGCCCAATACAACAGATCACGTG
R_URA3_BE	G*GAATTCGGATCC*AGGTTCTATCGAGGAGAAAAAGCG
F_ERG1p_*Bam*HI	CG*GGATCC*GTCGAATACTACTATGACCG
R_ERG1p_*Eco*RI	G*GAATTC*CCAATTGTAATAGCTTTCCCATGACCCTTTTCTCGATATGTT
F_ERG11p_*Bam*HI	CG*GGATCC*CTTGTTCTCTCTCGCTTCC
R_ERG11p_*Eco*RI	G*GAATTC*CCAATTGTAATAGCTTTCCCATCCTTGTATTACTCGTTTGTTC
F_ERG2p_*Bam*HI	CG*GGATCC*AGTGTTAGCAAGCGCAGACG
R_ERG2p_*Eco*RI	G*GAATTC*CCAATTGTAATAGCTTTCCCATGGCTATAATGGTCTGGGCTAG
F_ERG3p_*Bam*HI	CG*GGATCC*GAATATCGTCAACCTCGTCC
R_ERG3p_*Eco*RI	G*GAATTC*CCAATTGTAATAGCTTTCCCATATCTCAAATCTAGACGAATATT
F_ADS_*Bam*HI	CG*GGATCC*AAAACAATGGCCCTGACCGAAGAG
R_ADS_*Xho*I	ACACG*CTCGAG*TCAGATGGACATCGGGTAAAC
F_ERG9p_Int	GGTTTTGGGTTTAGTGCCTAAACGAGCAGCGAGAACACGACCACG
R_ERG9p_Int	CTTCATCTCGACCGGATGCAATGCCAATTGTAATAGCTTTCCCAT
Primer for qPCR study	
F_ACT1_q	TCCGTCTGGATTGGTGGT
R_ACT1_q	TGAGATCCACATTTGTTGGAAG
F_ERG1_q	TGTTGGTGCCAAGGTTGA
R_ERG1_q	AAATGTCAAGTGGGCTTTGAA
F_ERG11_q	TGCACGTTCCAAACACTTCT
R_ERG11_q	CCTGGAGAAACCAAAACGTG
F_ERG2_q	AATTGGCTCAAGGCTGGATT
R_ERG2_q	TGGAGAAAGTGTCCAAAAACC
F_ERG3_q	CAACTACGGTCAATTCACCACTC
R_ERG3_q	AATGAGTCATCTGGTCTACGGTAA
F_ERG9_q	TTACAATTGGCATTGCATCC
R_ERG9_q	TTCTGCAAAACTTCAGCTTCAA

For generating the mutant strains with *ERG9* under the control of different ergosterol-responsive promoters, electroporation was carried out as follows. Fresh overnight culture of strain M4-2nd was inoculated into 50 mL YPD medium to an initial OD_600_ 0.3. Yeast cells were harvested by centrifugation at 4°C, 1500 g for 5 min after 4–5 h when OD_600_ reached 1.3. The cell pellet was washed twice with 50 mL ice-cold Milli-Q water, followed by centrifugation to collect the cells. Next, the cells were washed with 4 mL ice-cold 1 M sorbitol, pelleted by centrifuge and finally re-suspended in ice-cold sorbitol to a final volume of 400 μL. Subsequently, 50 μL of yeast cells together with approximately 2 μg of genome integration cassette were electroporated in a 0.2 cm cuvette at 1.6 kV. After electroporation, cells were immediately mixed with 2 mL pre-warmed YPD medium and shaken for 90 min on a rotary shaker to recover the cells. Cells were spotted on SC-URA plates and incubated at 30°C for 3–4 days until colonies appeared. Strains with successful replacement of native *ERG9* promoter were verified by diagnostic PCR.

Since previous investigation showed that low copy expression of *ADS* gene resulted in higher amorpha-4,11-diene titers [28], centromeric plasmid based expression of *ADS* gene was created as follows. Briefly, *ADS* gene was amplified from pRS425ADS, cut with *Bam*HI/*Xho*I and inserted into pSH68 cut with the same enzyme pair, to yield pRS415ADS. For the transformation of pRS415ADS into engineered strains, the standard lithium acetate method was used and the transformed cells were spotted on SC-LEU plates for selection.

### RNA extraction and quantitative real-time PCR

Yeast cells were harvested at early-log phase and total amount of 1 × 10^7^ cells was used for the total RNA extraction using the RNeasy Mini Kit (QIAGEN, Germany). Approximately 500 ng of RNA was converted to cDNA using iScript^TM^ Reverse Transcription Supermix from Biorad (Hercules, CA).

The gene-specific primers for *EGR9*, *ERG1*, *ERG11*, *ERG2*, *ERG3* and *ACT1* were designed using the ProbeFinder (https://lifescience.roche.com), and oligonucleotides used for qRT-PCR experiment were listed in Table 3. Quantitative PCR analysis was carried out using LightCycler 96 real-time machine with FastStart Essential DNA Green Master Mix (Roche) according to the manufacturer’s instructions. Each 20 μL reaction contained 50 ng of total cDNA, 10 μL FastStart Essential DNA Green Master Mix, 0.5 μM of each primer. Thermal cycling conditions were set as follows: pre-incubation, 1 cycle of 95°C for 10 min; amplification, 45 cycles of 95°C for 10 s, 57°C for 10 s and 72°C for 10 s. *ACT1* was chosen as a reference housekeeping gene and the results were presented as ratios of gene expression between the *ERG9*, *ERG1, ERG11, ERG2, ERG3* and the reference gene, *ACT1* [29].

### Amorpha-4,11-diene production in engineered yeast

To investigate the effect of ergosterol-responsive promoters on restricting *ERG9* expression in the engineered strains, both the growth profile and amorpha-4,11-diene production profile were investigated. Strains harboring pRS415ADS were inoculated into SC-LEU medium. The next day, 250 mL flasks containing 25 mL SC-LEU medium (1.8% galactose + 0.2% glucose) were inoculated with fresh cell cultures to an initial OD_600_ of 0.05. All flasks were immediately supplemented with 20% (vol/vol) dodecane after seeding, to perform two phase fermentation and harvest amorpha-4,11-diene. The growth profile was continuously monitored for 4 days. The amorpha-4,11-diene levels were measured after 48 h or 96 h cultivation. Every time, 100 μL of cell culture was taken for measuring OD_600_ by microplate reader (Synergy H1, BioTek, USA), and 10 μL dodecane layer was sampled and diluted in 990 μL ethyl acetate for the quantitation of amorpha-4,11-diene levels using gas chromatography-flame ionization detector (GC-FID). For GC-FID analysis, 1 μL of diluted sample was injected into Shimadzu QP2010Ultra system equipped with a DB-5 column (Agilent Technologies, USA). Hydrogen was used as a carrier gas at a flow rate of 1.0 mL/min. The oven temperature was first kept constant at 80°C for 2 min, and then ramped to 190°C at a rate of 5°C/min, and finally increased to 300°C by 20°C/min. For the quantitation of amorpha-4,11-diene levels, caryophyllene was used for plotting the standard curve and the results shown in the present study are presented as caryophyllene equivalents.
